# Compared Block Periodized and Non-Periodized Physical Activity Programs in Older Adults

**DOI:** 10.3390/sports12050119

**Published:** 2024-04-28

**Authors:** Alejandro Moreno-Mateos, Fausto José Barbero Iglesias, Antonio Sánchez Muñoz, Yurena Gutiérrez Díaz, Carlos Moreno Pascual

**Affiliations:** 1 Department of Sports, Salamanca City Council, 37003 Salamanca, Spain; 2 Department of Nursey and Physiotherapy, University of Salamanca-IBSAL, 37007 Salamanca, Spain; fausbar@usal.es; 3 Education College, Pontificia University of Salamanca-EGIIOFID, 37007 Salamanca, Spain; asanchezmu01@upsa.es; 4 Health Service of the Canary Islands, 35508 Las Palmas, Spain; yurenagutierrez80@gmail.com; 5 Department of Nursey and Physiotherapy, University of Salamanca, 37007 Salamanca, Spain; moreno@usal.es

**Keywords:** physical activity, older adults, periodized training

## Abstract

Background: The periodization of physical exercise to optimize objectives is common in competitive sports. However, physical exercise programs for older adults only sometimes present periodization in their programming. Therefore, this article aims to research the results of applying the sports periodized method to older adults. Methods: A total of 137 participants over 60 years old performed a physical exercise program; 71 participated in a multi-component non-periodized program as the Control Group (CG), and 66 participated in a program periodized in blocks as the Experimental Group (EG). The block periodization program was oriented to the development of strength and was carried out in 86 sessions thrice weekly for eight months. Anthropometric assessments were made using weight, height, Body Mass Index, and electrical bioimpedance; and functional evaluations were made through standardized tests: Short Performance Physical Battery (SPPB), Timed Up & Go (TUG), handgrip, and a two-minute stair test. Results: After the intervention, the EG significantly improved TUG, weight, and BMI. On the other hand, the CG showed significant improvements in fat weight, BMI, and the 2 min stair test. The SPPB did not show changes after the intervention. Conclusion: The periodization of physical exercise for older adults does not significantly impact functional capacity in this population group.

## 1. Introduction

Physical exercise is a critical factor in health. Its benefits for the older population have been widely documented, and the World Health Organization (WHO) [1] includes it in its health recommendations. Physical exercise programs for older adults are widespread, and various physical activities are offered.

The hypothesis of this study is that a block periodization model improves anthropometric and functional variables in older adults. The objective of this study is to apply a block periodized model to the older adult population as well as a non-periodized program in order to analyze the impact of both models on functional and anthropometric results and to define an 8-month periodized model.

Physical exercise must be carried out regularly, continued over time, and applied to long-term programs. For this reason, medium- and long-term goals must be established in order to enhance the beneficial effects of physical exercise and avoid the harmful ones.

Sport periodization is defined as the “intentional sequencing of different training units so that the athlete can achieve the desired state and planned results” [2]. It has been used since 1950 in high-performance sports in order to ensure that all sessions aid in reaching a stated goal. However, let us look at studies of physical exercise for older adults. It is common for a program’s performance to be assessed over a medium and long term because they have greater benefits.

Periodized physical exercise takes place jointly in other groups [3,4], while in the older population models based on the American College Sports of Medicine (ACSM) [5], indications for strength training are usually presented. It is rare to find research that describes a period. The standard study design is an ACSM strength model or similar. Bolam et al. [6] analyzed research about exercise in older adults, including any manuscript that included periodized exercise.

For example, a professional athlete’s season lasts between 9 and 12 months, and is usually periodized, during which they have moments of evaluation through competitions and a rest period. Similarly, many physical exercise programs for older adults are developed for 8 to 9 months, or even shorter, followed by a rest period that usually coincides with summer. Given the similarity of months dedicated to both high-performance sports and exercise for older adults, the idea arises to apply medium-term planning methods for sports, microcycles, mesocycles, and macrocycles to an older adult population.

The block periodization model group’s sessions had similar physical and technical objectives during the determined period of time, creating a progression in the subjects’ physical capacity and reaching a maximum point of fitness in a specific time. This model creates three blocks: one focused on the volume of work performed, a second directed at the intensity with which the exercise is performed, and the last one focused on whether the exercises are related to what will be evaluated [2].

In addition, the periodization in blocks has focused on strength training, so it is related to the periodization presented by the ACSM [5]. The non-periodized model is based on the Geriatric Revitalization Program (PReGe) [7] of the University of Salamanca, which is oriented towards multi-component physical development, with a low- or medium-intensity session structure, widely proven during the years of the program’s existence

## 2. Materials and Methods

### Participants

Inclusion criteria—Participation was voluntary via registration with the Salamanca City Council in the Geriatric PreGe. Participants were over 60 years of age, of both sexes, independent, autonomous, and non-institutionalized, from the municipality of Salamanca (Spain). 

Exclusion Criteria—A percentile lower than 15 in the two-minute stair test together with a relative contraindication or physical limitation; an absolute contraindication for physical exercise; presented a pathology that did not allow the normal development of the program; or did not carry out the evaluations. 

Groups—The PReGe was carried out in groups in different spaces in the city. This study included 137 people. They were distributed into the CG (Control Group) and the EG (Experimental Group) through cluster sampling. The EG was made up of 70 people, of whom four were excluded according to criteria, who carried out a program of physical exercise structured in blocks and oriented to the development of strength. The CG comprised 88 people, of whom 17 were excluded according to the criteria.

**Figure 1:**
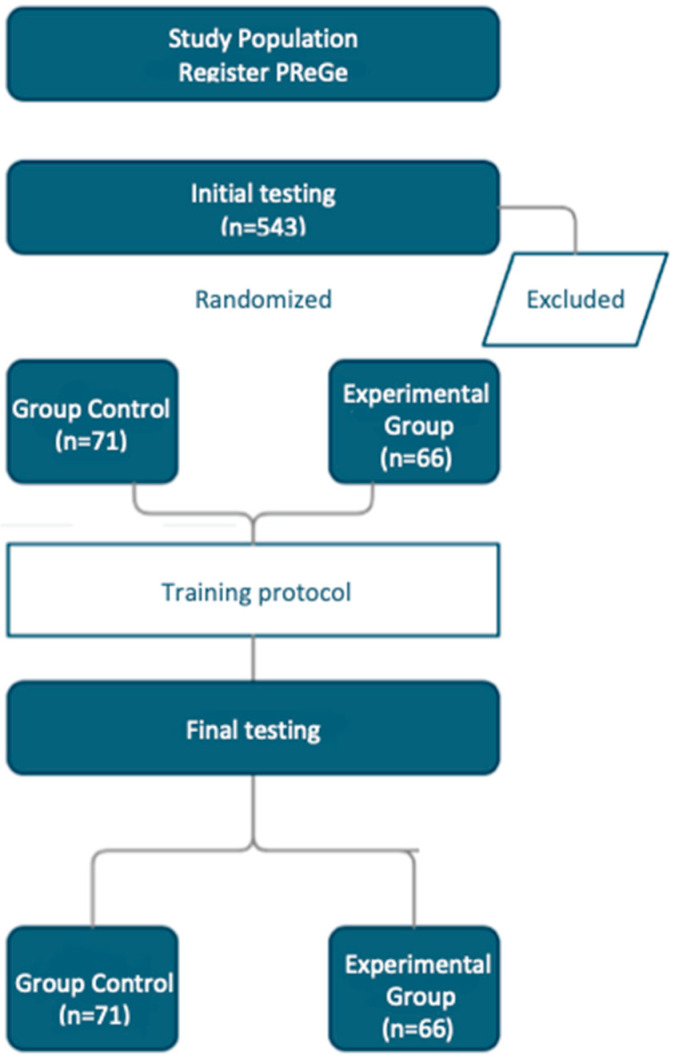
Randomized experimental research flow-chart.

Assessment and equipment—Two assessments were carried out, one prior to the intervention (E1) and another at the end of the intervention (E2) (Figure 1). This study was developed by the Faculty of Nursing and Physiotherapy of the University of Salamanca. The assessment consisted of anamnesis, anthropometric assessment, and functional assessment. The anthropometric assessment consisted of measuring weight using a scale, PPW3300/01 (Bosch, Stuttgart, Germany); foot size by stadiometer, SECA213 (SECA GMHB & Co, Hamburg, Germany); Body Mass Index (BMI); and the percentage of body fat assessed by electrical bioimpedance, OMROM BF 300 (Omrom Healthcare, Kioto, Japan). The functional assessment consists of the Short Physical Performance Battery (SPPB), which includes assessment of bi-standing balance, semi-tandem and tandem, displacement of 4 m, and strength of the lower limb, in which the participants were instructed to stand up and sit down as quickly as possible from a chair five times without using the arms; the Timed Up & Go test, in which the participants had to stand up from a chair, move to a mark located 3 m away, and return to sit in the chair; the Handgrip test by handgrip JAMAR Plus+ (Performance Health, Notts, UK); and the two-minute staircase of the senior fitness test [8], in which the participants had to raise their knees to hip height for two minutes alternately. Physiotherapists and a physical educator carried out the evaluations.

**Figure 2:**
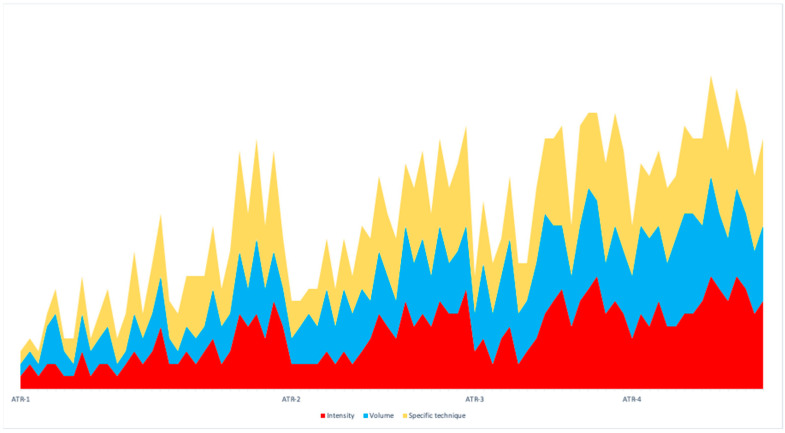
Graph of volume, intensity, and specific technique in block periodized.

**Figure 3:**
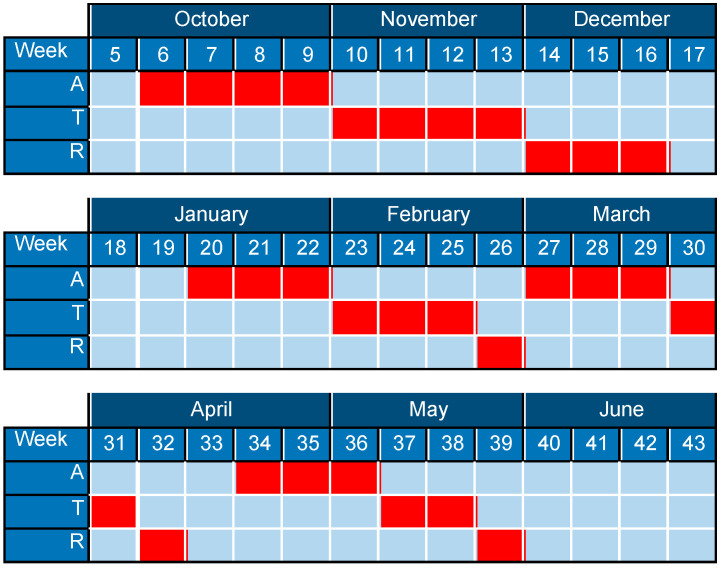
Gantt Chart about block periodization.

Intervention design—Two groups were proposed: an EG with the periodized intervention and a CG without periodization. The periodization of the EG was adjusted to three blocks per grouping, and in turn, four groupings of three blocks were made, with a total of 86 sessions of fifty min for eight months, with three sessions a week. The first block of each group was oriented to volume, the second to intensity, and the third to exercises similar to the tests contained in the evaluations. The session had a classic warm-up section, with low-intensity, low-impact joint mobility exercises; a central part, with submaximal strength and resistance-strength exercises; and a return to calm, with low-impact, low-intensity aerobic-resistance exercises. The volume block concentrated on the function of actions with more than 15 repetitions and a low-speed execution. The intensity block had less than 15 repetitions and a high-speed execution. In eight months, they performed four macrocycle ATRs (Figure 2 and Figure 3). For the CG, the intervention consisted of a PreGe [7] based on multi-component physical exercise with protocolization of the session. Each session includes static stretching, easy walking or running, breathing exercises, dynamic stretching and muscle strength exercises, easy walking or running, hydration, coordination and balance, easy walking or running, relaxed breathing, and hydration. A total of 86 sessions of 50 min were carried out for eight months. The University of Salamanca’s Bioethics Committee approved both groups’ interventions.

Statistical analysis—The statistical analysis was conducted using the IBM SPSS Statistics software, version 24 (SPSS v.24.0.0.0, IBM Corporation, New York, NY, USA). The means, medians, variances, standard deviations, minimums, and maximums were obtained, and the Kolmogorov–Smirnov normality test (Lillefors correction) was performed, obtaining a non-normal distribution. Homogeneity between groups was analyzed using the Krustal–Wallis H test. The analysis of the pre-and post-intervention differences used the Wilcoxon signed-rank test, which also analyzed by age ranges: under 75 years, between 75 and 85 years, and over 85 years. Due to the non-homogeneity between groups, an ANCOVA was carried out on the covariates of sex and age. A confidence interval of 95% and size effect α > 30 was taken.

## 3. Results

The results were analyzed based on a baseline after 86 sessions of intervention (Table 1 and Table 2), diving the population into three age ranges: under 75 (Table S1 for EG and Table S2 for CG), between 75 and 85 (Table S3 for EG and Table S4 for CG), and over 85 (Table S5 for EG and Table S6 for CG). 

The CG presented a significant weight reduction (*p* < 0.001) in its fat weight (*p* < 0.005), while the EG presented a significant weight reduction (*p* < 0.005). However, it is not clinically relevant in any group. The results of both groups improved significantly in terms of BMI. The calculation made by the fat percentage analysis system did not detect variation in the fat percentage in the EG. 

The results of the balance tests of the SPPB did not show a deterioration or an improvement after the intervention, for either the CG or the EG. The 4 m displacement time of the SPPB did not show significant losses or improvements for either group. The SPPB lower limb strength test did not show significant variations in the CG and EG. The strength of the upper limb test presented significant differences after the intervention for the EG. The 2 min stair test presented better results in the EG but without statistical significance, while the CG presented a significant improvement in results after the intervention (*p* < 0.005). For the Up & Go test, the EG presented a significant improvement (*p* < 0.005), while the CG did not present a change after the intervention. It should be noted that the <75 years subgroup of the CG presented a significant improvement in the 2 min stair test. For people in the range of 75–85 years, the CG presented a significant improvement in the 2 min stair test, while the EG presented a significant improvement in the Up & Go test.

Regarding the >85 years, the CG presented a significant improvement in the Up & Go test, as did the EG; the EG also presented a significant improvement in the strength of the left upper limb test.

## 4. Discussion

This study aimed to apply a sport periodization model to older adults by comparing it with another model. However, the non-homogeneity of the random register in the groups did not permit us to compare the groups. Nevertheless, it can be confirmed that a periodized model helps an older adult exercise program to improve different areas of physical condition (or at least, to maintain physical condition) at E2.

The periodization models of physical exercise derived from high-level competition sports are difficult to adapt to the characteristics of the older population, so it was decided that the experiment would be conducted using a physical exercise program with a long history, PReGe [7], and a widely contrasted sports training model, the ATR, in order to discover if the latter could provide additional benefits compared to the study population that participated in the traditional program.

Regarding the results of the anthropometric variables, the BMI improved from E1 to E2, putting both results in the overweight condition of the BMI classification [9] for both the CG and the EG. After the intervention, the EG and the CG, in all age groups, presented a BMI reduction, as seen in other studies relating physical exercise to BMI [10,11].

Body weight presented a statistically significant reduction in both groups, but is not clinically relevant. On the contrary, a study by Ruiz-Montero [12] observed that weight was maintained or even increased in older adults with a 24-week program of aerobic Pilates, the same as Villareal [13], who concluded that physical exercise should be accompanied by a caloric restriction diet so that there is a reduction in weight. The Waters [14] review suggests that weight loss through physical exercise in people over 65 is possible if accompanied by a change in lifestyle. It should be taken into account that the process of muscle atrophy accelerates after the age of 50, and can reach a loss of 30% of muscle mass by the age of 80 [15], which influenced our evaluation—the older a study population is, the more it will be affected. The sarcopenia process can be retained or slowed down by strength training, with the EG model being the most beneficial for treating the pathology [16].

The results obtained for fat weight reduction are consistent with previous studies; physical activity enables the control and reduction of adipose levels [17,18]. The CG presented a significant reduction in fat weight. The EG presented a statistically significant reduction in weight, although not in fat weight. From this, it can be concluded that the EG model is not recommended for the maintenance of lean mass, since a decrease in total body weight has been observed, but without a decrease in fat mass.

The results of the SPPB balance assessment tests did not show any variation in either of the two groups (CG and EG) participating in this study. In contrast, previous PReGe studies did show improvements in balance [19], although other studies suggest that specific programs to improve balance [20] should be implemented in order to achieve positive results. The literature [21,22] suggests that multi-component physical exercise models improve the results of lower limb strength tests, but, paradoxically, those that have a more significant impact on the strength component present more modest results or show no variation. Multi-component physical exercise programs have been presented as a method to improve handgrip strength [23]. The CG presented good results in the hand grip strength test (handgrip) since it maintained the values after the intervention, which is relevant from the point of view of avoiding deterioration inherent to aging. It is noteworthy that the EG presented significant improvements in the handgrip test after the intervention in those over 85 years of age. The results obtained in the two-minute stair test vary according to age stratification, but studies such as those by Michael [24], Taguchi [25], and Severinsen [26] suggest that physical exercise programs present improvements in aerobic tests. Severinsen [26] indicated that in physical exercise programs with a high predominance of strength work, specific tasks are required to improve in the six-minute walk test.

The results of our study suggest that the two models of physical exercise (CG and EG) are valid for the older population to maintain physical and functional capacity since no negative evolution was observed in the tests carried out. For its part, a multi-component physical exercise model is more suitable for improving aerobic capacity. On the other hand, the strength-oriented physical exercise model presented a better result in strength-velocity, evaluated through the TUG, mainly in the group over 75 years of age.

Interventions that do not propose standardized planning presented the effects produced by physical exercise at a random point within the program, rather than at the end of a cycle, as with a periodized program, making it difficult to compare the results of one intervention or another. The EG planning model of our study does not conform to the classic methods of periodization of physical exercise in the older population (linear, non-linear, and unplanned) [27]. For this reason, no studies have been published that fit models similar to this.

The SPPB semi-tandem and feet-together test results did not change after the intervention. The tandem test did not present significant variations in either of the two groups; however, it maintained the results obtained over time, which is positive for the study population. The systematic review by Latham et al. [20] indicates that physical exercise programs focused mainly on the development of strength do not significantly influence the results obtained in balance. Hafström [28] verified that there could be an improvement in balance in eight weeks with a proposal similar to the CG, while other research suggests that multi-component groups only affect dynamic balance [29]. The study by Patil [30] with 409 women between 70 and 80 years of age found similar results, mainly in the SPPB, for 12 months of physical exercise similar to the CG. The review by Cadore and Rodríguez Mañas [31] also concluded that physical exercise improves balance. However, it is chiefly the multi-component programs that present improvements in this aspect, and specific exercises should be included to improve this quality. The EG did not present notable variations in the results of the balance assessments in our study, but it did show a positive impact by maintaining the results over time. As previously mentioned, the implementation of a physical exercise program relevant to strength training, regardless of the periodization chosen, is not a model indicated for improving balance [32,33].

Our study did not find a statistically significant improvement in the 4 m walk test in any of the groups, but it did maintain the results, avoiding deterioration caused by age. In most cases, the differences between the means of E1 and E2 are tenths or hundredths of a second; these differences could be due to the observation of the evaluator, and therefore, in future studies, it should be collected using electronic systems, such as photoelectric cells. The improvement of gait speed by a multi-component low-intensity physical exercise program, such as that of the CG, is presented in different studies, such as the review and meta-analysis conducted by Hortobàgyi [34]. The study by VanSwearingen et al. [35], with 47 participants with a mean age of 77.2 years, concluded that multi-component training improves movement speed. However, better results are presented with a program more focused on gait re-education. Studies suggest that the development of force through non-linear periodization models allows improvements in short-distance displacement [36,37]. Our study found that walking speed capacity for short distances was maintained through a force-oriented ATR periodization. Bårdstu et al. [38], in a study with 104 participants, found that the results of gait speed for 20 m stabilized in the group oriented to strength training after eight months. As previously indicated, Severinsen [26] considers it necessary to include specific gait activities to improve this indicator. In contrast, Cadore [31] does not consider it necessary to include this type of exercise to improve gait speed.

In our study, the lower limb strength test, corresponding to the SPPB, did not present significant variations, the same as in the 4 m test. However, there are studies [39,40] where multi-component physical exercise models present improvements in lower limb strength. The study conducted by Oreskà et al. [22] with nineteen 65-year-old subjects (±3.62) found significant improvements in the results obtained by a multi-component exercise program. The lower limb strength test tends to be used to analyze power, which is why it is usually used in studies that include a program to develop strength [41]. The results of these studies may be derived from the fact that the samples were sedentary, while our population had been participating in PReGE over the years. The study by Bårdstu [38], cited above, concluded that a strength-oriented physical exercise program significantly improved lower extremity strength test results. However, Cadore et al. [31] concluded that using a multi-component or force model shows no improvement in lower extremity strength. On the other hand, the ATR approach of the EG of this study is positive in lower extremity strength since it maintains the results, although other studies present improvements in the mean results of this test [42]. On the other hand, Conlon et al. [43] point out that the use of a block or linear periodization model does not make a difference to the study result in this test.

Studies that present multi-component programs have shown positive results in assessing upper limb strength [38,39]. In the present work, the results obtained by the CG were maintained after the intervention, avoiding deterioration caused by age. Training models focused on strength can effectively improve the results in the upper limb test [43]. Our study confirms that a strength-oriented program, such as the EG, maintains, and tends to improve, the average results in the upper limb. However, there is no significant difference.

The CG showed a positive improvement in the two-minute stair test. Lichtenstein et al. [44] concluded in a study with 27 people that a multi-component intervention presented better results than a traditional strength program, as seen in other studies [45]. The update carried out by Cadore and Izquierdo [46] suggests that the programs focused on the development of strength and aerobic capacity are the ones that present the best results in tests to assess aerobic capacity in a submaximal exercise such as the 2 min stair test. Murlasits and Reed [47] hypothesized that non-linear strength-oriented training does not affect the results of a test such as the 6 min walk or, failing that, the 2 minute stair test, as has been observed in the EG of this study.

Lacroix et al. [48] and Zhuang et al. [49] found that a multi-component physical exercise program such as that of the CG improves the results of the TUG test. In our study, the CG did not present better results, but did maintained them; this is relevant in order to avoid deterioration in the studied population. The TUG test presented a statistically significant improvement in the EG, consistent with the observations of Coelho-Júnior et al. [50] in a study with 45 women (60–79 years old), where the use of a DUP periodization model for 23 weeks decreased the completion time of the test by more than 50%. The significant improvement in this population is noteworthy, mainly in those over 85 years of age, considering that these programs aim to maintain capability or avoid physical deterioration.

The effects of the different interventions must be taken as independent since there is no homogeneity in E1, and therefore, the groups are not comparable. The results obtained in our study, as Strohacker [51] already observed, do not allow us to definitively confirm that block planning is more effective than a non-periodized model.

The multi-component program showed improvements for aerobic capacity, body weight, and fat weight. In contrast, the periodization program for strength training showed improvements for strength speed, an indicator of fall prevention.

The first limitation of this study is the non-homogeneity between the two groups; future studies must control the conformity of the groups. Other study limitations should note that Christmas and Easter stopped the program for a few days, which could result in detraining. Additionally, each group was managed by different people, which could affect the involvement of the participants. Finally, managing the volume and intensity using no direct systems, like pulsimeters, could alter the results.

Future studies should apply two multi-component programs, one periodized and the other non-periodized. They should also use an intensity and volume system with direct control, like pulsimeters or other methods, and analyze the effects of stopping the program for days in older people.

## 5. Conclusions

We cannot conclude that a periodized intervention in blocks is more effective than a non-periodized intervention, but rather that, as has been widely documented, physical exercise, in any of its proposed forms, is beneficial for older adults, since it improves or maintains functional capacity. However, periodization makes it possible to improve the efficiency of physical exercise programs for older adults, providing them with a progression.

## Figures and Tables

**Table 1:** Baseline and Results of Experimental Group.

Experimental Group	Baseline E1 (N = 66)	Final E2 (N = 66)	*p*-Value
Characteristics			
% Women	57.6%		
Age average (years)	79.05 (0.665)		
Anthropometric			
Weight (kg)	65.7711 (1.74292)	66.1833 (2.11625)	0.005 *
Height (cm)	152.7211 (1.09042)		
BMI (kg/m^2^)	28.1172 (0.59470)	28.0718(0.74608)	0.005 *
% Body Fat	43.505 (0.6764)	43.250(0.7466)	0.425
Fat weight (kg)	28.676 (0.9276)	28.720 (1.1319)	0.073
Functional Test			
Tandem (s)	9.4150 (0.29859)	9.3940 (0.33264)	0.313
4 m Walking (s)	4.4976 (0.21932)	4.4757 (0.219221)	0.100
Strong leg (s)	9.9895 (0.51394)	9.5557 (0.48254)	0.335
Handgrip Right (kg)	20.2611 (0.80943)	21.7367 (1.04495)	0.131
Handgrip Left (kg)	20.0237 (1.07427)	21.0277 (1.12509)	0.133
Up & Go (s)	9.1147 (0.41202)	8.0890 (0.41591)	<0.001 *
2′ Step (steps)	788,684 (3.72874)	81.5333 (3.72526)	0.706

Results present the mean and standard deviation (in parenthesis). “*” marks the significant differences and a medium-sized effect. BMI—Body Mass Index.

**Table 2:** Baseline and results of Control Group.

Control Group	Baseline E1 (N = 71)	Final E2 (N = 71)	*p*-Value
Characteristics			
% Women	80.3%		
Age average (years)	76.94 (0.709)		
Anthropometric			
Weight (kg)	67.6047 (1.79252)	67.6282 (1.61937)	<0.001 *
Height (cm)	152.6279 (0.90962)		
BMI (km/m^2^)	28.9689 (0.69109)	28.8882 (0.65808)	<0.001 *
% Body Fat	43.258 (0.7936)	42.833 (0.8653)	0.365
Fat weight (kg)	29.726 (0.9977)	29.136 (1.0432)	0.004 *
Functional Test			
Tandem (s)	8.7426 (0.44153)	9.3210 (0.30898)	0.122
4 m Walking (s)	4.1860 (0.08964)	4.2777 (0.11265)	0.907
Strong leg (s)	10.6198 (0.30173)	10.5118 (0.34213)	0.132
Handgrip Right (kg)	20.7907 (0.87883)	19.1949 (0.58231)	0.445
Handgrip Left (kg)	20.3209 (0.94277)	26.8359 (4.88899)	0.812
Up & Go (s)	8.1479 (0.31960)	7.8069 (0.18312)	0.132
2′ Step (steps)	78.0698 (1.556243)	82.0256 (2.55849)	<0.001 *

Results present the mean and standard deviation (in parenthesis). “*” marks the significant differences and a medium-sized effect. BMI—Body Mass Index.

## Data Availability

The datasets presented in this article are not readily available because personal data. Requests to access the datasets should be directed to University of Salamanca.

## Supplementary Materials

The following supporting information can be downloaded at https://www.mdpi.com/article/10.3390/sports12050119/s1. Table S1: Baseline and results of Experimental Group under 75 years. Table S2: Baseline and results of Control Group under 75 years. Table S3: Baseline and results of Experimental Group between 75–85 years. Table S4: Baseline and results of Control Group between 75–85 years. Table S5: Baseline and results of Experimental Group over 85 years. Table S6: Baseline and results of Control Group over 85 years.
