# Supplementary material for: Compared Block Periodized and Non-Periodized Physical Activity Programs in Older Adults
Source: Sports (Basel). 2024 Apr 28;12(5):119. doi: 10.3390/sports12050119 (PMC11125595; doi:10.3390/sports12050119)
Supplement: Supplementary file 1 [file sports-12-00119-s001.zip › sports-2942130-supplementary.pdf]

Content

1. eTable 1. Baseline and results of Experimental Group under 75 years 2
2. eTable 2. Baseline and results of Control Group under 75 years 3
3. eTable 3. Baseline and results of Experimental Group between 75-85 years 4
4. eTable 4. Baseline and results of Control Group between 75-85 years...................5
5. eTable 5. Baseline and results of Experimental Group over 85 years 6
6. eTable 6. Baseline and results of Control Group over 85 years...............................7

eTable 1. Baseline and results of Experimental Group under 75 years

Experimental Group <75 years	Baseline (N=12)	Session 86 (N=12)	p-value
**Characteristics**			
**% Women**	33,3%		
**Age average**	71,83 (,737)		
**Anthropometric**			
**Weight**	65,7333 (4,27337)	64,3333 (4,58863)	,054
**Height**	151,1000 (2,49663)		
**BMI**	28,5633 (1,39803)	27,9453 (1,58093)	,041*
**% Body Fat**	44,111 (1,2988)	43,678 (1,2770)	,463
**Fat weight**	28,856 (1,7841)	28,433 (1,6898)	,208
**Functional Test**			
**Tandem**	8,8438 (,78422)	8,2675 (1,11670)	,273
**4m Walking**	4,4513 (,20566)	4,6975 (,10001)	,754
**Strong leg**	9,7763 (,72894)	9,2613 (,89642)	,272
**Handgrip Right**	19,0250 (1,35419)	19,4750 (2,50704)	,965
**Handgrip Left**	20,7125 (3,73270)	20,0125 (2,50866)	,575
**Up & Go**	76,1250 (5,17700)	80,8750 (5,34335)	,061
**2’ Step**	8,4275 (,51695)	7,8013 (,33642)	,695

eTable 2. Baseline and results of Control Group under 75 years

Control Group <75 years	Baseline (N=28)	Session 86 (N=28)	p-value
**Characteristics**			
**% Women**	85,7%		
**Age average**	71,00 (,491)		
**Antrhopometric**			
**Weight**	65,7333 (4,27337)	68,1481 (1,81220)	,18
**Height**	151,1000 (2,49663)		
**BMI**	28,5633 (1,39803)	29,4940 (,76992)	,017*
**% Body Fat**	44,111 (1,2988)	43,500 (,7068)	,409
**Fat weight**	28,856 (1,7841)	29,807 (1,0847)	,027*
**Functional Test**			
**Tandem**	8,8438 (,78422)	8,6513 (1,23975)	,050*
**4m Walking**	4,4513 (,20566)	4,2571 (,15881)	,428
**Strong leg**	9,7763 (,72894)	10,3714 (,72590)	,333
**Handgrip Right**	19,0250 (1,35419)	19,4429 (1,13756)	,882
**Handgrip Left**	20,7125 (3,73270)	19,2214 (,90628)	,665
**Up & Go**	76,1250 (5,17700)	83,1429 (2,17846)	,638
**2’ Step**	8,4275 (,51695)	7,6657 (,23104)	,005*

eTable 3. Baseline and results of Experimental Group between 75-85 years

Experimental Group 75-85 years	Baseline (N=43)	Session 86 (N=43)	p-value
**Characteristics**			
**% Women**	65,1%		
**Age average**	78,86 (,419)		
**Antrhopometric**			
**Weight**	68,0049 (1,73899)	67,6610 (1,72644)	,180
**Height**	153,7930 (,98593)		
**BMI**	28,5212 (,55330)	28,3821 (,55648)	,180
**% Body Fat**	42,641 (,7539)	43,071 (,7056)	,083
**Fat weight**	28,976 (,8803)	29,051 (,8937)	,706
**Functional Test**			
**Tandem**	9,5789 (,42105)	9,7158 (,17494)	,875
**4m Walking**	4,0537 (,23328)	4,1632 (,18536)	,244
**Strong leg**	9,7205 (,56642)	9,6121 (,55192)	,894
**Handgrip Right**	21,1011 (1,20604)	22,2632 (1,44064)	,346
**Handgrip Left**	20,5684 (1,34582)	21,2175 (1,50248)	,293
**Up & Go**	82,6842 (5,35306)	79,7895 (5,46952)	,020*
**2’ Step**	8,5226 (,44443)	7,4805 (,31243)	,645

eTable 4. Baseline and results of Control Group between 75-85 years

Control Group 75-85 years	Baseline (N=31)	Session 86 (N=31)	p-value
**Characteristics**			
**% Women**	74,2%		
**Age average**	78,77 (,470)		
**Antrhopometric**			
**Weight**	68,0049 (1,73899)	68,2708 (2,07796)	,012*
**Height**	153,7930 (,98593)		
**BMI**	28,5212 (,55330)	29,1837 (,81803)	,041*
**% Body Fat**	42,641 (,7539)	42,763 (1,2878)	,403
**Fat weight**	28,976 (,8803)	29,213 (1,2919)	,072
**Functional Test**			
**Tandem**	9,5789 (,42105)	8,0481 (,72464)	,638
**4m Walking**	4,0537 (,23328)	4,0781 (,14719)	,427
**Strong leg**	9,7205 (,56642)	10,4895 (,37667)	,192
**Handgrip Right**	21,1011 (1,20604)	20,3524 (,76577)	,171
**Handgrip Left**	20,5684 (1,34582)	20,5810 (,78415)	,717
**Up & Go**	82,6842 (5,35306)	81,6667 (4,50256)	,784
**2’ Step**	8,5226 (,44443)	8,0914 (,28293)	,000*

eTable 5. Baseline and results of Experimental Group over 85 years

Experimental Group >85 years	Baseline (N=11)	Session 86 (N=11)	p-value
**Characteristics**			
**% Women**	54,5%		
**Age average**	87,64 (,801)		
**Antrhopometric**			
**Weight**	59,4091 (2,59003)	58,4364 (2,63222)	,038*
**Height**	151,5636 (2,03641)		
**BMI**	25,8203 (,85590)	25,3855 (,86343)	,038*
**% Body Fat**	44,091 (1,1935)	44,018 (1,3127	,756
**Fat weight**	26,473 (1,0890)	25,536 (1,2181)	,016*
**Functional Test**			
**Tandem**	8,8533 (1,14667)	7,6550 (1,48311)	,068
**4m Walking**	5,5467 (,93106)	5,4567 (,87487)	,213
**Strong leg**	10,5050 (2,74410)	10,6950 (1,42325)	,203
**Handgrip Right**	19,2833 (2,93841)	18,5333 (1,39968)	,075
**Handgrip Left**	17,7000 (1,65630)	18,8833 (1,21392)	,041*
**Up & Go**	67,3333 (14,14842)	80,8333 (5,37536)	,028*
**2’ Step**	11,8567 (1,59458)	10,0217 (1,66375)	,540

eTable 6. Baseline and results of Control Group over 85 years

Control Group >85 years	Baseline (N=12)	Session 86 (N=12)	p-value
**Characteristics**			
**% Women**	83,3%		
**Age average**	86,08 (,313)		
**Antrhopometric**			
**Weight**	59,4091 (2,59003)	61,6364 (2,99628)	,021*
**Height**	151,5636 (2,03641)		
**BMI**	25,8203 (,85590)	26,6227 (1,21648)	,028*
**% Body Fat**	44,091 (1,1935)	43,827 (1,4983)	,593
**Fat weight**	26,473 (1,0890)	27,127 (1,8607)	,423
**Functional Test**			
**Tandem**	8,8533 (1,14667)	9,0100 (,69932)	,674
**4m Walking**	5,5467 (,93106)	4,9338 (,26390)	,084
**Strong leg**	10,5050 (2,74410)	11,3338 (,87792)	,638
**Handgrip Right**	19,2833 (2,93841)	18,3125 (1,30267)	,410
**Handgrip Left**	17,7000 (1,65630)	19,4500 (1,05526)	,388
**Up & Go**	67,3333 (14,14842)	81,3750 (3,18443)	,350
**2’ Step**	11,8567 (1,59458)	7,9538 (,43513)	,036*
